# Post COVID-19 Transformation in the Frequency and Location of Traffic Crashes Involving Older Adults

**DOI:** 10.1177/03611981231163866

**Published:** 2023-05-04

**Authors:** Ali Soltani, Mohammad Azmoodeh, Mohsen Roohani Qadikolaei

**Affiliations:** 1 Injury Studies, Flinders Health and Medical Research Institute, Flinders University, Bedford Park, SA, Australia; 2 Babol Noshirvani University of Technology, Babol, Iran; 3 University of Guilan, Rasht, Iran

**Keywords:** before-and-after safety studies, crash analysis, crash frequency, safety performance, COVID-19, older adults

## Abstract

Although numerous studies have been conducted to discover the spatial patterns of road crashes, relatively few have focused on the patterns of road crashes suffered by socially disadvantaged groups, while simultaneously accounting for urban environmental features. This study used advanced econometric (negative binomial regression) and spatial (geographically weighted Poisson regression) approaches to capture latent geographical diversity in crash patterns. The police-reported crash data for the over-65 population in metropolitan Adelaide, Australia, were investigated for two periods: before and after COVID-19. Using both spatial and nonspatial models, the effects of land use mix, population density, road network design, distance to the central business district, and accessibility of public transit on crash frequency, and location at the neighborhood level were investigated. The findings revealed that, in addition to sociodemographic factors, the aforementioned components had nonlinear effects in varied geographical contexts. Although the number of crashes fell by 20% during the periods studied, the fundamental reasons for such incidents did not change. The results of the study could assist academics and policy makers in Australia to better understand the multidimensional implications of the built environment on the road safety of the elderly—a vulnerable group in society who were disproportionately affected by the global pandemic. The hybrid technique presented in this research has the potential to be useful in other scenarios experiencing varying crash patterns.

Every year, over 1.35 million people worldwide die in crashes involving motor vehicles, with vulnerable road users such as pedestrians, motorcyclists, and cyclists accounting for 54% of those deaths (*1*). The elderly are one of the demographics most susceptible to being involved in a car crash because they have decreased cognitive and driving skills, and their crash incidence and the severity of the incidents vary significantly from those of other age groups (*2*, *3*).

The over-65 population in Australia is forecast to grow from 16% in 2020 to 22% by 2050, whereas the number of over-80s is expected to increase from 4% to 8% (*4*). The aging population in Australia is therefore likely to increase car reliance among older people, as well as the number of older individuals holding a driver’s license, whereas public transportation usage is expected to decline (*4*). Between 2008 and 2021, the percentage of older persons (aged 65+) killed in car crashes in Australia grew significantly from 16% to 22%. In recent years, numerous countries (e.g., Canada) have discontinued older adults driver's license renewal programs, which require persons aged 80 and older to undergo vision, hearing, and cognitive screening exams as well as undergo a review of their driving histories (*5*).

## COVID-19: Changes in Travel Patterns and Crash Rates

With the outbreak of the COVID-19 pandemic, most governments were compelled to impose mandatory quarantines that resulted in the closure of educational institutions, businesses, as well as recreational, social, cultural, and leisure enterprises (*5*); work-from-home and staying home policies were also introduced. Overall, during COVID, the number of trips decreased worldwide (*6*). During the quarantine period, congestion in cities such as Paris, Milan, Manchester, and Madrid decreased by an average of 80% compared with the previous year (*7*). Furthermore, there was a transition in modal split. For example, the share of nonmotorized commutes to work grew significantly. However, the frequency of crashes involving cyclists also increased: the average number of cyclists who died or were injured in collisions more than doubled (*8*). Overall, the reduction in traffic has resulted in fewer road crashes and fatalities (*9*). A study in Spain showed that the number of traffic accidents fell by a large amount because of the steps taken to stop the pandemic (*10*). In Italy, the lockdown (in March and April 2020) and mobility restrictions triggered by COVID-19 led to a significant drop in car crashes (about 70%) and in the associated health problems they cause (*11*). Comparable research conducted in Greece indicated that road accidents dropped by 49% during the COVID-19 months compared with non-COVID-19 periods.

However, drivers’ habits were also observed to shift as a result of the decreased traffic volumes, notably in relation to average speed, speeding, and forceful braking per 100 km. Specifically, a higher-than-anticipated average increase in speed of 2.27 km/h was reported, along with an increase in severe braking/100 km of about 1.51 km (*12*). In several regions of the United States, the incidence of fatal and severe injury crashes increased. This phenomenon of increased speeding and riskier driver behavior was found to occur in conjunction with the decreased traffic levels. In relation to different driver cohorts, because of COVID-19 mobility limitations and the reduced kilometers traveled, some studies demonstrated that car injuries and pedestrian fatalities for older adults were fewer during the pandemic than before (*9*). It is currently unknown how COVID-19 has influenced crash severity, however crash research in New York and Seattle found that the decrease in transportation demand caused by the pandemic led to reduced crash frequencies (*13*).

COVID-19 may have reduced overall traffic levels, but it led to a rise in serious injury crashes in Alabama (*14*). A correlation between the pandemic and a rise in the probability of serious car crashes was also found in Virginia owing to changes in driving habits (*15*). Although daily vehicle miles traveled and Motor Vehicle Crash (MVC) decreased, single-vehicle fatal crash rates climbed by 410% compared with the pre-lockdown period in Connecticut, United States. According to police statistics, incidence of speeding was found to increase throughout the pandemic, indicating that people in Connecticut were more willing to take risks when driving (*16*). During the lockdown in North Carolina (*17*) and Virginia (*18*), the share of crashes attributable to high speeds also rose. Accidents leading to death or serious injury rose in Maine, in northeastern United States, during the COVID-19 stay-at-home order (April and May of 2020). Findings have also revealed that even 1 year after the stay-at-home order was issued speeding persisted, demonstrating the lasting effects of the changes on driver behavior (*19*). In the United States, counties that had “safer-at-home” orders saw an average of 1.4 crashes per day. The number of crashes before COVID-19 was 1.77 per day per county (*20*). However, although the frequency of crashes in New York City decreased in this period, their severity rose from 0.25 casualties per collision to 0.45, in a 3-month period (April to July 2020) (*8*). During the first week of the stay-at-home order in Connecticut, the incidence of single-vehicle accidents increased significantly (*21*). Crash rates began to recover to normal once the stay-at-home restriction was lifted. However, the fatal crash rate was unaffected by the stay-at-home mandate (*21*).

Although the COVID-19 lockdown resulted in a decrease in the incidence of crashes, it has been correlated with increased crash severity, acceleration, and severe recurrent braking crashes (*7*).

### Factors Correlating with Crash Frequency

Potential factors affecting the occurrence of crashes, particularly among older adults have been listed in research, including socioeconomic (e.g., education, income) and demographic characteristics (e.g., age, gender) (*22*), land use activities (24–26), alcohol and drug use, distraction, driving offenses, and behavioral disorders (*26*), and vehicle quality and its components such as the navigation system and brakes (*27*). Other road design elements include slopes, curves, crossings, and their horizontal and elevation changes; traffic signage, stop signs (lack or incorrect signage), and speed limits as part of implementing traffic regulations; lighting and microclimatic and environmental conditions; and built environment elements such as population distribution and density, land use dispersion, and road network layout patterns.

Older adults are frequently involved in crashes on inner-city arterials (*28*). Conflict and speed are associated with the risk of being exposed to crashes. Road and traffic safety measures have been applied to save lives by slowing the growth in road crash deaths in the face of an increasing population (*29*).

Population and employment density, as well as the variety of land uses, are aspects of the built environment that would be expected to affect travel behavior (*30*). A higher density of land uses is generally associated with lower walking distances being required to reach destinations. Walking is also associated with variations in land-use activities and the geometric structure of street networks, including the number of intersections (*31*) and the average block size (*32*). Older people are more sensitive to built-environment features, such as travel distance, owing to reducing physical strength and mental abilities. Conversely, policies that encourage land-use mix may indirectly reduce safety levels for older people by making them more exposed to vehicular traffic and the associated collision risk (*33*).

However, the association between the distribution of land-use activities and the incidence of crashes is ambiguous (*34*). Some studies have discovered a negative relationship between industrial zones and the occurrence of serious crashes, whereas other scholars have found a positive correlation in their investigations. Commercial strip operations along major arterials and mixed-use projects are positively related to crash rates, particularly those involving pedestrians, although the severity of the crashes might be lowered by decreasing traffic speeds (*35*). Commercial, industrial, and office land-uses may increase the growth of pedestrian- and vehicle traffic volumes, but residential land-uses may reduce car–pedestrian crashes (*36*). In other words, the built environment has a larger effect on crash deaths than individual factors (*37*).

Suburban neighborhoods have higher crash rates and are viewed as being less safe than urban districts owing to the residents’ reliance on cars, higher roadway capacities (i.e., number of lanes), higher vehicle speeds, and the use of large vehicles resulting from urban dispersion (*38*). The volume of traffic attracted to certain places will be determined by the policies governing the placement of, for example, megaprojects, major trip generators, and the development of urban highways, thus resulting in higher rates of traffic crashes (*34*). In some studies, freeways were found to generate fewer fatal crashes than other facilities owing to their higher design standards and better access control (*39*).

As a result of increased activity and conflict between pedestrians and vehicle traffic, increasing the ratio of commercial/office areas, road network densities, and the counts of designated intersections might increase the likelihood of pedestrian crashes (*40*, *41*). Although intersections can reduce traffic speeds by obstructing off-straight routes to deviate traffic flow from the direct path, they can increase the permeability of the network, thus increasing through-traffic (*42*). The presence of regional arteries and other types of roads, the number of vehicle lanes and routes, and other street traffic characteristics such as the frequency and density of intersections in various forms, as well as the length of the sidewalk and the presence of public transportation stations, can have relationships with car and pedestrian collisions (*29*, *36*).

Crash frequency is connected to sociodemographic attributes such as age, car ownership, family size, and -income, as these factors affect both the origin of the travel, where people live, and the purpose of travel, because they are related to their characteristics and lifestyle choices (*43*). For example, in the case of pedestrian crashes, research has investigated socioeconomic inequities in pedestrian and built-environment safety. Deprived areas usually lack adequate pedestrian facilities, increasing the risk of pedestrian crashes. Although affluent regions may have a higher supply of highways, traffic safety measures, equipment, and interventions, suburban districts are typically labeled as less safe for nonmotorized transport (*36*, *44*). Individuals in higher-income communities are expected to be able to afford safer vehicles, thus, are less exposure to risky conditions. In contrast, residents in disadvantaged neighborhoods are likely to face higher risks of traffic crashes owing to a higher share of nonmotorized commuting or use of less safe vehicles (*44*).

Furthermore, there is a substantial, positive association between population density and fatal crashes, with the aging rate being a significant factor (*45*), because for the older pedestrian, there is a higher crash risk owing to reductions in age-related cognitive ability (*46*). Data have also revealed that the likelihood of collisions reduces with a larger household, because older adults are frequently accompanied by younger family members. However, the proportion of those aged 18 and less is positively associated with fatal crashes, because the young are more likely to take greater risks while traveling (*38*). It was found that an increase in the share of older cohorts (75+) was correlated with fewer crash counts (*47*). However, the results can conflict between contexts, as neighborhoods with higher shares of older adults have been found to correlate with higher traffic crashes (*48*).

## Conceptual Model

**Figure 1.**
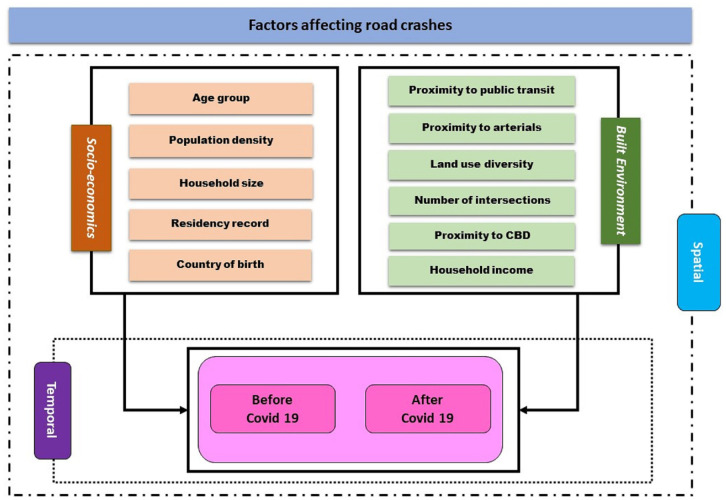
Conceptual model. Note: CBD = central business district.

The conceptual model considers two types of factors that influence crash frequency on a before-and-after comparison: socioeconomics (including demographics) and household characteristics. Here, we focused on five characteristics that have been demonstrated to have a substantial relationship with crash frequency: age group, household income, household size, residence record, and birth country. The second type includes built-environment aspects comprising six major variables: proximity to public transit, proximity to arterials, diversity of land use, number of intersections, proximity to central business district (CBD), and population density (Figure 1). The model investigates both spatial and temporal aspects.

The contribution of this research is to provide a methodology for better understanding the crash patterns of older adults based on a combination of sociodemographic and built-environment attributes drawn from existing datasets on a fine-grained scale, which is block-wide. Previous literature considered the general public, but this study focused on a vulnerable portion of society: those aged >65. This study investigated the influence of location as a moderator of crash-causing elements on a local scale, as opposed to the worldwide scale examined in previous studies.

The study addressed the extent to which road crash frequency and spatiotemporal patterns among older adults have transformed following the adoption of COVID-19 restrictions. Specifically, this research addressed the following three questions:

1. What are the frequency and severity effects of COVID-19 on the incidence of crashes involving older individuals in South Australia?
2. Do the impacts of the crash-causing factors (built-environment and socioeconomic) before and after COVID-19 differ?
3. How does the influence of geography differ on crashes before and after the pandemic?

This is the first time a large-scale safety analysis of Adelaide’s suburban structure has been conducted in relation to how well it engenders safety for older adults.

## Methodology

### Negative Binomial Regression Model

Since crash counts are nonnegative discrete data that usually deviate from normal distribution, Poisson- and negative binomial regression (NBR) are the most common methods used to model crash frequency (*49*). Of these, in cases of an overdispersed dataset (i.e., variance > mean), NBR is preferred over the Poisson model (assumes variance = mean). Because the overdispersion test at the 1% significance level showed that the crash data variance exceeded the mean, and the crash count data were the dependent variables for both models, NBR was selected. The mathematical expression of NBR to model occurred crashes is as follows:

$$\mathrm{Pr}\left(Y=y_{i}|\mu_{i},\alpha \right)=\;\frac{\Gamma \left(y_{i}+\alpha^{-1}\right)}{\Gamma \left(y_{i}+1\right)\Gamma \left(\alpha^{-1}\right)}{\left(\frac{1}{1+\alpha \mu_{i}}\right)}^{\alpha^{-1}}{\left(\frac{\alpha \mu_{i}}{\alpha^{-1}+\alpha \mu_{i}}\right)}^{y_{i}}$$

where *y_i_* is total crashes, α is the overdispersion parameter, and *μi* is the expected frequency of crashes in block *i*.

### Geographically Weighted Poisson Regression

In a geographically weighted Poisson regression (GWPR) model, the frequency of crashes is determined by a set of explanatory factors that may vary in space (Table 1). Consequently, when spatial nonconstant relationships appear, the estimated coefficients will be the function of ( $u_{i},v_{i}$ ), which supplies the geographic coordinates of the centroid of the *ith* census block. The equation can be written as follow (*50*):

$$\ln\left(\lambda_{i}\right)=\beta_{0}\left(u_{i},v_{i}\right)+\beta_{1}\left(u_{i},v_{i}\right)\sum_{k}\beta_{k}\left(u_{i},v_{i}\right)x_{\mathrm{ik}}$$

where *λ_i_* is the expected frequency of crashes in block *i*, and *β_k_* (*u_i_,v_i_*) represents the coordinates of the geographical center of block *i*,

**Table 1.** Descriptive Statistics for Variables

Variable*	Sociodemographic					Built environment						Before_countcrash	After_countcrash
	Age group	Effect of CBD and income together	Household size	Residency record	Country of birth	Proximity to public transit	Population density	Proximity to arterials	Land use diversity	Intersections	Proximity to CBD		
	Over 65 years	CBD_Income (km*$)	Average household size	5 years resident	Born overseas pop.	Dist. to PT (km)	Population density (population/ha)	Dist. to major roads (km)	Land use mix	Count intersections	Dist. to CBD (km)		
Abbreviation	pop_65+	cbd_incm	hh_sz	res_rcrd	bp_ovrsz	dst_pt	pop_dns	dst_mrds	lum	cnt_intrsc	dst_cbd		
Definition	Number of adults over 65	Distance to CBD multiplied by weekly median income of block (in Australian dollars)	Average number of household members	Proportion of residents with five years of residency record	Number of persons born overseas	Distance between centroid of block and nearest public transportation station	Population divided by area	Distance between block and major roads (AADT > 24,000)	Entropy index of land use categories within the block	Frequency of intersections within the block	Distance between centroid of block and CBD		
Mean^†^	71.46	17.8	2.37	58.18	175.66	1.156	367.44	1.196	0.475	61.44	13.986	0.862	0.388
SD	47.93	13.203	0.56	33.2	86.72	2.299	291.92	1.743	0.165	67.631	9.104	1.538	0.811
VIF^§^	2.873	2.927	2.07	2.117	2.534	2.981	2.654	2.46	1.113	1.282	2.213		

Note: CBD = central business district; Dist. = distance; PT = public transport; pop. = population; AADT = annual average daily traffic.

* For all variables, *N* = 3,016.

† The average population of each Australian census block (SA1) = 400 people.

§ As a rule of thumb for correlation between predictors of a model. VIF = 1: no correlation between, 1 < VIF < 5: moderate correlation, and VIF > 5: potentially severe correlation.

$$\hat{\beta }\left(u_{i},v_{i}\right)={\left(X^{T}W\left(u_{i},v_{i}\right)X\right)}^{-1}X^{T}\left(u_{i},v_{i}\right)Y$$

where

$\hat{\beta }$ (*u_i_,v_i_* ) = vector of estimated coefficients in block *i*,

*X* = matrix of exogenous factors,

*Y* = vector for the dependent variable (DV), and

*W*(*u_i_,v_i_*) = matrix of geographic weight.

$$W\left(u_{i},v_{i}\right)=\left[\begin{array}{cc} \begin{array}{cc} w_{i1} & 0\\ 0 & w_{i2} \end{array} & \begin{array}{cc} \cdots & 0\\ \cdots & \; \end{array}\\ \begin{array}{cc} \cdots & \cdots \\ 0 & \cdots \end{array} & \begin{array}{cc} \cdots & \cdots \\ \cdots & w_{\mathrm{ij}} \end{array} \end{array}\right]$$

where *w_ij_* = weight of variable *j* in location *i*.

A GWPR is based on the idea that the data points in close spatial proximity to the regression line have greater influence in predicting the coefficients than those at further distances. Therefore, the effect of this proximity would be applied by choosing a weighting function kernel and its bandwidth. There are two types of weighting functions: Gaussian and bi-square.

Gaussian:

$$w_{\mathrm{ij}}=\exp\left(-\frac{1}{2}\times {\left(\frac{d_{\mathrm{ij}}}{b_{i}}\right)}^{2}\right)$$

Bi-square:

$$w_{\mathrm{ij}}=\left\{ \begin{array}{c} {\left[1-{\left(\frac{d_{\mathrm{ij}}}{b_{i}}\right)}^{2}\right]}^{2}\;d_{\mathrm{ij}}<b_{i}\\ \;0\;\mathrm{otherwise} \end{array}\right.$$

where

*w_ij_* = spatial weight of *jth* block on *ith* block,

*d_ij_* = Euclidean distance between blocks *i* and *j*, and

*b_i_* = kernel neighborhood (bandwidth).

As Fotheringham et al. stated, the bandwidth is more important than the type of kernel, because kernel bandwidths control the distance decay in *w_ij_* and an increase in kernel width may lead to missing local variations (*50*).

## Data

**Figure 2.**
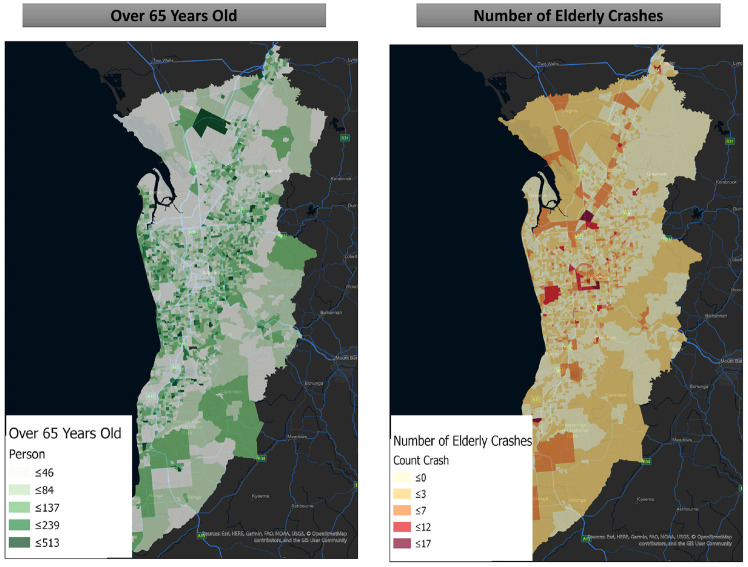
Map of metropolitan Adelaide: distribution of older adults versus distribution of crashes.

Australia’s demographic landscape is changing with a rapid increase in the aging population: 15% of Australians (equivalent to 3.8 million people) were 65 and above in 2017. This proportion is expected to rise significantly over the next few decades, with 12.8 million individuals (25%) reaching the age of 65 or older by 2097 (*51*). South Australia has the highest proportion of older people in mainland Australia, comprising more than 37% of the total population. Adelaide, with a land area of 3,259.8 km^2^, is the capital of South Australia, and the fifth-most populous city of Australia (more than 1.4 million residents) (*52*) (Figure 2).

A set of sociodemographic and urban data and Police-reported crash data was collated from secondary sources, including the Australian Bureau of Statistics. This study focused on older adult (65+) group data, which comprise a smaller share than children and young adolescent (<15 years) and working-age populations (aged 15 to 64) in Australia (*51*). For the sociodemographic- and crash analysis of this research (sample size > 3,000), Statistical Area One (SA1) was used since this is the smallest viable geographical unit for analysis in the census data. SA1 has a permanent population of around 447. Over a 2-year period, SA1-level crash data were received from the state government (2019 to 2021). The data were collected over the course of 2 years: 1 year before and 1 year during COVID-19.

On February 25, 2020, the first incidence of COVID-19 in Australia was recorded. The nation has recorded 5,162,934 cases and 20,758 fatalities since then (*53*). Australia ranks 126th in the overall number of COVID-19 cases and fatalities worldwide. It is widely considered to have been one of the most effective nations in the fight against the pandemic (*54*). One of the most effective initiatives was the implementation of travel restrictions on both internal and international mobility, which resulted in a 98% drop in travel in April 2021 compared with April 2019 (*43*). To curb the spread of COVID-19, the government banned all nonessential travel (e.g., an banned social gatherings) and reduced public transportation in mid-March 2020. This dramatically decreased the use of public transportation and private cars. In April 2020, public transportation use fell by 46%, visits to recreational locations fell by 23%, and just 15% of people continued to commute to work (*43*). In metropolitan Adelaide, the frequency of total road crashes reduced by approximately 7%, traffic volumes dropped by 8%, injury crashes decreased by 18% during lockdowns, but the causing factors remained the same as pre-COVID 19. The hotspots for crashes shifted from work and education areas to green spaces and city fringes, and the frequency of crashes changed from weekdays to weekends and winter to summer (*35*).

Additional steps included limiting the number of persons who could attend public meetings, and banning nonessential indoor and outdoor gatherings. Following this, visitors and personnel to elderly care facilities were restricted, and social gathering spots and recreation spaces were closed. In addition, social distancing measures were implemented, there were delays to all nonurgent elective surgeries, working from home was encouraged, and internet shopping increased (*55*). As an illustration of the result of these efforts, Australia saw record online shopping figures, climbing to 41% in April 2020, up from 17% at the same time in 2019 (*43*).

The researchers used regression models to examine the frequency of accidents involving older people before and after the implementation of COVID-19 restrictions, with the frequency of accidents as the dependent variable. In the regression models, the frequency of accidents involving older people, which is a discrete number, was a dependent variable (DV). This variable was tested twice: before COVID-19 (years 2017, 2018, and 2019) and after (in 2020). Because COVID-19 restrictions were implemented on March 23, 2020 (*56*), the remainder of the year (9 months) was taken into account. It should be noted that *N* = 1,802 is three-quarters of the average number of crashes in the period 2017 to 2019, representing 9 out of 12 months. The selection of 3 years of data was motivated by the need to have more observations and increase the accuracy of the data. In addition, at the start of this research, only 9 months of data were obtainable. Therefore, we selected the 9-month period of 2020 to represent post-COVID-19, and compared this with the average 9-month data from the preceding 3 years (pre-COVID-19). The explanatory variables were a continuous mix of socioeconomic and urban structural aspects that are believed to affect the frequency of crashes. Only the most influential components were chosen, while checking for collinearity among the variables analyzed. The variance inflation factor (VIF) was utilized for this purpose. Table 1 describes the statistically significant components and their respective descriptive statistics.

## Results

### Patterns of Crash, Pre and Post COVID-19

Table 2 compares the proportion of factors involved for all crash records before and after COVID-19 in Adelaide, Australia; among these, certain factors showed significant differences (*p* < 0.05) in their contribution to the crash counts. Generally, the total number of postpandemic crashes (*N* = 1,450) decreased by 19.5% compared with the same period before the pandemic (*N* = 1,802; chi-square = 37.873, *p* < 0.05), which was a result of the reduced number of high-risk conflicts caused by the application of COVID-19 lockdown rules and travel restrictions in the city.

**Table 2.** Details of crashes involving older adults (65+), pre and post COVID-19

Characteristics	Before (*N* = 1,802)		After (*N* = 1,450)		*p*-Value*
	*N*	%	*N*	%	
Gender					.524
Male	1,100	61.0	901	62.1	NA
Female	702	39.0	549	37.9	NA
Crash zone					.195
CBD	72	4.0	54	3.72	NA
Metropolitan area	1,421	78.9	1,175	81.0	NA
Regional area	309	17.1	221	15.24	NA
Days of week					.672
Sunday	157	8.71	139	9.59	NA
Monday	257	14.26	208	14.34	NA
Tuesday	307	17.04	234	16.14	NA
Wednesday	316	17.54	247	17.03	NA
Thursday	288	15.98	223	15.38	NA
Friday	298	16.54	243	16.76	NA
Saturday	179	9.93	156	10.76	NA
Monthly					≈ 0
April	204	11.32	103	7.10	NA
May	234	12.99	141	9.72	NA
June	205	11.38	144	9.93	NA
July	211	11.71	181	12.48	NA
August	196	10.88	171	11.79	NA
September	177	9.82	172	11.86	NA
October	194	10.77	186	12.83	NA
November	193	10.71	173	11.93	NA
December	188	10.43	179	12.34	NA
Light condition					.027
Daylight	1,543	85.6	1,280	88.3	NA
Night	259	14.4	170	11.7	NA
Crash severity					.650
PDO	1,032	57.3	842	58.1	NA
MI	611	33.9	461	31.8	NA
SI	133	7.4	125	8.6	NA
Fatal	26	1.4	22	1.5	NA
Older adults’ responsible					.524
Yes	1,075	59.66	849	58.55	NA
No	727	40.34	601	41.45	NA
Alcohol and drugs					.765
DUI involved	18	1.00	13	0.90	NA
Drugs involved	17	0.94	13	0.90	NA
Traffic controls					.788
Give way sign	184	10.2	150	10.3	NA
No control	1,128	62.6	900	62.1	NA
Other	1	.1	1	.1	NA
Rail xing: boom	1	.1	1	.1	NA
Rail xing: flashing	1	.1	1	.1	NA
Roundabout	82	4.6	61	4.2	NA
Stop sign	104	5.8	67	4.6	NA
Traffic signals	301	16.7	269	18.6	NA
	Mean	SD	Mean	SD	*p*-Value*
Age	74.10	7.099	73.93	6.814	.496
Area speed limit	62.52	16.526	73.933	6.813	≈ 0

Note: CBD = central business district; DUI = driving under the influence; Rail xing = rail crossing; PDO = property damage only; MI = mild injury; SI = serious injury; NA= not available.

* Pearson’s chi-squared significance test was employed to compare proportions. Two-sample *t*-tests were used to compare means. Statistical significance was set at *p* < 0.05.

We are aware that although a 20% reduction in the number of crashes was observed, the underlying causes remained largely same. Nonetheless, as stated in the manuscript, the purpose of this research was to offer a hybrid approach (Poisson + Geographically Weighted Regression) that could be more relevant in other contexts with varying crash patterns.

Concerning the distribution of records on weekdays, the data indicated that the share of crashes on busy working days of the week (e.g., Monday, Tuesday, and Wednesday) reduced. With the introduction of COVID-19, the closure of many businesses, and the dramatic increase in employees working remotely, traffic volumes and the percentage of crashes on these days reduced.

Therefore, considering the share of crashes over months, the first months of the pandemic in South Australia (i.e., April [4.2%], May [3.3%], and June [1.5%]) showed a significant decrease compared with the similar period before the pandemic. The results indicated that lockdown restrictions and business closures appeared to have reduced traffic and, naturally, the crash counts in the first 3 months after COVID-19 (*7*).

Lighting conditions showed a significant difference between the two periods: after the pandemic, the share of crashes during daylight hours increased, which was mainly a result of the restrictions applied to nightlife venues.

Following the pandemic, a significant number of crashes have occurred in places with higher speed limits. COVID-19 changed the social lives of people around the world, such that, with spatial distancing orders, people tended to spend their leisure time outside cities to avoid crowds—the main source of disease transmission (especially for older adults). Consequently, since regional areas of the city usually have higher speed limits, this difference indicated that crashes after the pandemic mostly occurred in these areas. The rate of crashes resulting in severe casualties (SI) also increased, which demonstrates that although the number of crashes reduced after the pandemic, the percentage of total crashes that resulted in more severe casualties increased owing to lower traffic volumes and the propensity of individuals to drive faster (*7*).

### NBR Modeling

This section will describe the generalized linear model that was used to examine the correlation between the number of crashes that occurred in each of Adelaide’s 3,016 census blocks before and after the pandemic.

The results of the NBR models for two defined conditions are presented (Table 3). NBRs used the log of the expected crash counts as a function of the regressors to model the DVs. Table 3 presents the NBR coefficient (*β*) along with its significance for 0.001, 0.01, and 0.1 levels, in addition to the incident rate ratio (IRR) that indicates the predicted change in DVs for a one-unit increase in the predictors. Generally, the results of the omnibus test for both models (*M_b_* = 769.061***, *M_a_* = 387.326***) indicated that the current NBR models outperformed the null model, and showed improvement over the baseline model. Moreover, overall goodness-of-fit reports (AIC_*Mb*_ = 7011.065 > AIC_*Ma*_ = 4599.494), (BIC_*Mb*_ = 7083.205 > BIC_*Ma*_ = 4671.634) revealed the model for after the pandemic (*M_a_*) was better fitting than that of the before pandemic.

**Table 3.** NBR Modeling Results, Pre and Post COVID-19

Variables		Mb		Ma		
	*β*	*t*-statistics	IRR	*β*	*t*-statistics	IRR
Constant	0.469**	–2.563	1.599	–0.279	–2.253	0.757
Sociodemographics						
5 years resident	–0.006***	–4.947	0.994	–0.006***	–4.223	0.994
Born overseas	0.002***	4.153	1.002	0.002***	3.339	1.002
CBD_income	–0.021**	–3.370	0.980	–0.012	–1.704	0.988
Over 65 years	0.007***	6.531	1.007	0.006***	4.961	1.006
Average household size	–0.114***	–2.642	0.886	–0.150***	–2.281	0.850
Built environment						
Population density	0.001***	7.541	1.001	0.001***	5.688	1.001
Land use mix	0.483***	4.476	1.417	0.484***	3.623	1.420
Distance to PT	0.082***	3.328	1.085	0.056*	1.843	1.058
Count intersections	0.004***	8.410	1.004	0.003***	5.836	1.003
Distance to CBD	–0.016*	–2.389	0.984	–0.017*	–2.054	0.983
Distance to major roads	–0.238***	–7.535	0.788	–0.195***	–4.967	0.823
Omnibus test	769.061***			387.326***		
Akaike information criterion	7,011.065			4,599.494		
Bayesian information criterion	7,083.205			4,671.634		
Degrees of freedom	3,004			3,004		
Number of observations (*N*)	3,016			3,016		

IRR = incident rate ratio; PT = public transport; CBD = central business district.

Dependent variable: number of crashes in each census block before pandemic (M_a_) and after pandemic (M_b_).

* *p* < 0.1; **p < 0.01; ***p < 0.001.– refers to minus sign.

“Landuse Mix (lum)” had a substantial positive influence on the frequency of crashes for *M_b_* (i.e., crashes in each census block after the pandemic) and *M_a_* (i.e., crashes in each census block before the pandemic), indicating that more land-use mixedness led to a larger number of crashes in the blocks, whether before or after the pandemic. The frequency of crashes rose in these places because pedestrian and vehicle conflicts were exacerbated by the presence of traffic-generating activities, and because of the physiological decreases in the decision-making process that come with advancing age (*36*, *57*). In addition, as a physically and functionally integrated aspect of CBDs, mixed land-use zones can lead to cognitive and visual distractions and therefore increase the likelihood of errors by older persons in complicated settings with high levels of conflict (*35*). On-street parking was found to increase the likelihood of dangerous collisions because it disrupts traffic flow and diverts drivers’ attention away from where they need to be looking. This was additionally associated with the impact of “Population Density (pop_dns)” and “Distance to CBD (dst_cbd)” on crash frequency. Within the census blocks with a greater density of population (*β_Mb,Ma_* = 0.001***) but closer to CBDs (*β_Mb_* = –0.016*, *β_Ma_* = –0.017*), where traffic congestion was caused by the workers of CBDs and the high cost of parking fees, more crashes happened pre and post COVID-19. Moreover, the behavior of the “CBD_Income (cbd_incm)” (*β_Mb_* = −0.016*, *β_Ma_* = −0.017*) variable, showed that neighborhoods with more well-off populations located in the outer suburbs correlated with a lower crash frequency. Not only do these individuals often dwell farther from CBDs, but also have more disposable income, which suggests they may have increased their use of delivery services during the pandemic (*45*).

Another effective regressor was “Average Household Size (hh_sz),” which had a significant inverse relationship with crash rates both before and after the pandemic (*β_Mb_* = –0.114***, *β_Ma_* = –0.150***), suggesting that a larger average number of individuals who live in a residence reduced the likelihood of crashes involving the elderly, largely because adults over 65 years of age tend to depend on younger family members to take care of some of the routine activities, such as shopping; this therefore decreases vehicle accessibility and consequently exposure to crashes. Slight increases in the *β* coefficient also indicated that since the risk of severe illness from COVID-19 was greatest for older adults, after the pandemic, the dependency on other family members and crash counts also increased and decreased, respectively. Because this was an aggregated study based on the census block, it is likely that average household size played a proxy role for certain sociodemographic variables that were missed in the analysis.

When looking at the built environment, “Distance to major roads (dst_mrds)” was the second noteworthy variable that had an effect on crash rates: in Adelaide, living closer to minor arterial roads led to more crashes (*β_Mb_* = −0.238***, *β_Ma_* = −0.195***). Both perception reaction times (PRTs) and the incidence of crashes involving older individuals are likely to rise significantly as a result of factors such as a relatively high speed limit, at-grade intersections with adjacent routes, and sections that enable bikes and pedestrians to traverse the road. Consequently, the influence of this variable considerably decreased (by about 18%) owing to the pandemic lockdown and remote working, which encouraged individuals to remain at home. Similarly, the rise in interactions between “collision units” (i.e., cars, bikes, pedestrians) explains the large positive connection between crash counts and the “Count Intersection (cnt_intrsc)” factor. Results from the “Over 65 years (pop 65+)” variable, where the *β* coefficient was substantially positive for both models, corroborated the study’s goal with respect to low PRTs for the older people category (*β_Mb_* = 0.007***, *β_Ma_* = 0.007***). Because of this, areas that included a greater number of seniors also tended to have more crashes, both before and after the pandemic.

There were significantly fewer crashes near public transportation stations, as shown by the results of the model predictor “Distance to PT (dst_pt)” (*β_Mb_* = 0.082***, *β_M_*_a_ = 0.056*). It all comes down to people’s need for security and the typical travel patterns for older people, for two reasons: 1) many public transit terminals are strategically located in lower speed limit zones, or in areas where traffic-calming measures are in place, and 2) because older adults are less likely to drive and more likely to take public transportation (*58*) the neighborhoods around stations serve as conflict-free havens for that age group. The magnitude and significance of *β* in *M_a_* lessened as a result of the pandemic, causing individuals, particularly the elderly, to avoid using public transportation.

Additional sociodemographic factors were also found to be important in both models: “5year_Resident (res_rcrd)” and “Born Overseas Population (bp_ovrsz).” Census blocks where the percentage of long-term residents was higher are safer overall, according to the “5year_Resident (res_rcrd)” results (*β_Mb_* = −0.006***, *β_Ma_* = −0.006***). To rephrase this, having lived at least 5 years in a given area increased environmental knowledge about the high-risk spots where traffic collisions have historically been concentrated, and helped older adults to decrease PRTs, enabling them to predict potential conflicts in certain areas and avoid collisions by making appropriate decisions. However, this was not the case for “Born Overseas Population (bp_ovrsz)” (*β_Mb_* = 0.002***, *β_Ma_* = 0.002***): those who were born outside of the United States but had adopted its driving norms and ethos were more likely to engage in risky behavior and be at fault in more crashes.

#### Hotspot Locations

**Figure 3.**
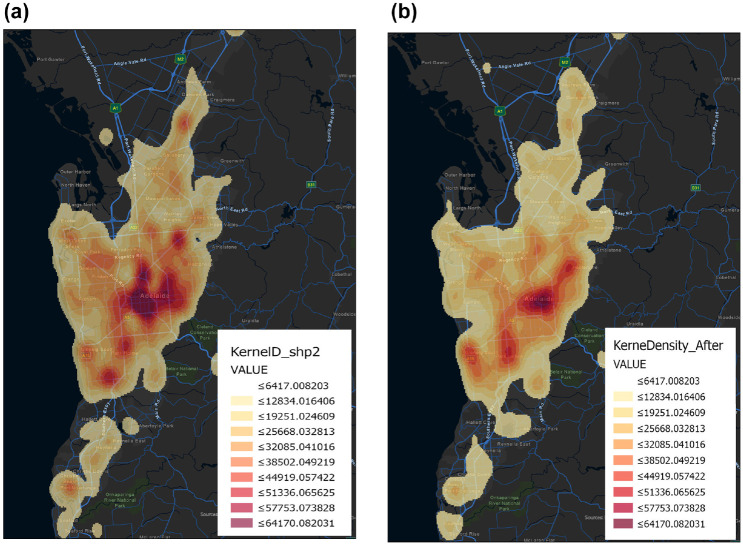
Kernel density estimation of crash frequency for older adults: (*a*) before and (*b*) after COVID-19.

The Kernel Density tool calculates the density of features in a neighborhood around those features. It can be calculated for both point and line features. The kernel model produced a map showing the locations of high-crash zones for the elderly in the metropolitan area, however, these places are constantly changing. In certain ways, the concentration of crashes within the city center, western, and northern wards weakened after COVID-19, whereas hotspots in the eastern and southern wards remained unchanged (Figure 3).

### GWPR Modeling

**Figure 4.**
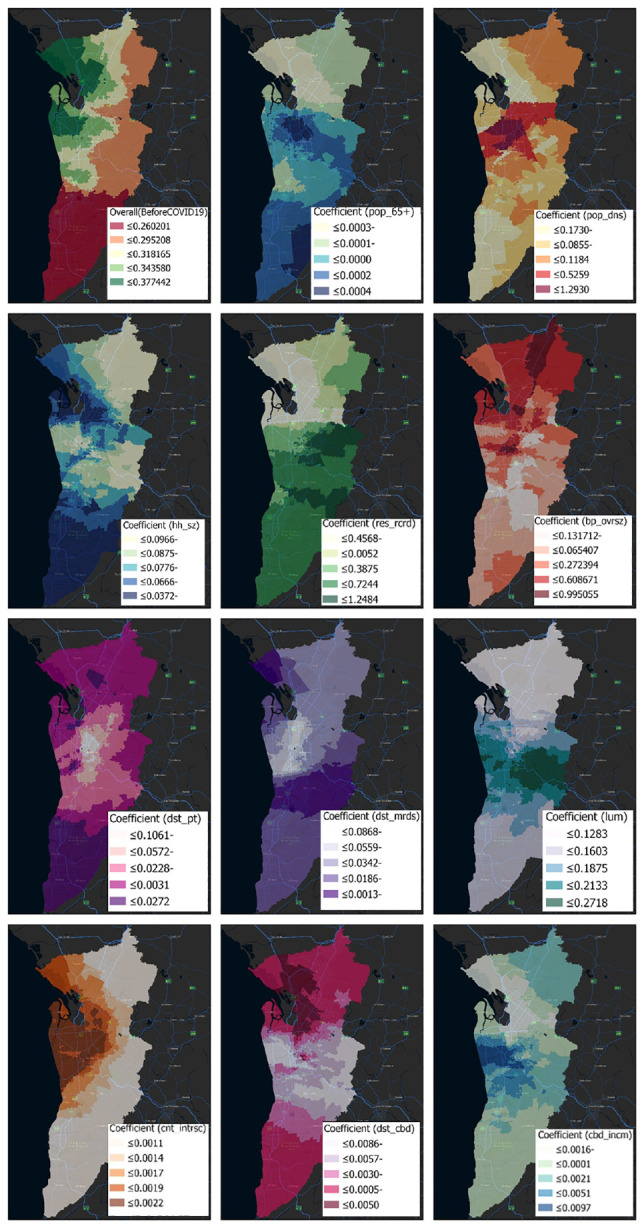
Coefficients of factors affecting older adults’ crashes (before COVID-19).

**Figure 5.**
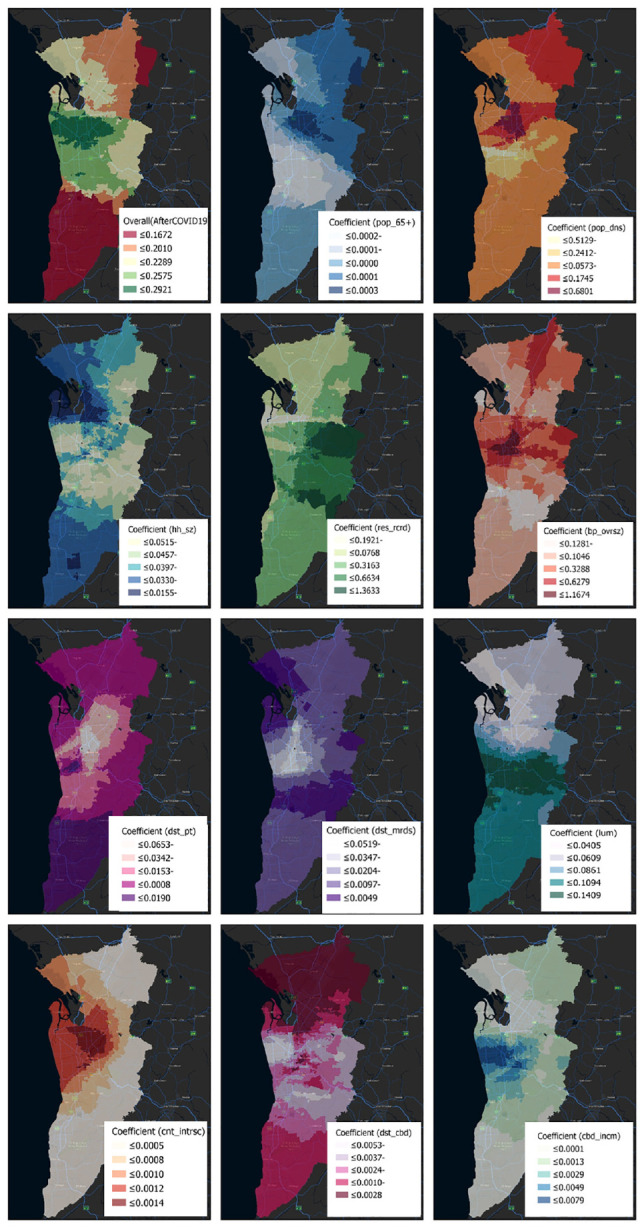
Coefficients of factors affecting older adults’ crashes (after COVID-19).

Unlike the NBR model, which gives a unique estimation for each variable, the variable estimates for GWPR models differ throughout space and can have surprising indications (Figures 4 and 5) (*60*).

Each independent variable displayed geographically evident patterns of change. The CBD and northwest (between Salisbury and Osborne) are key employment centers, and so the influence of most factors was greatest there. The coefficients for certain variables (i.e., over 65 years, population density, average household size, born overseas, distance to major roads, land use mix, count intersection) were compatible with the NBR model, whereas others were not.

For the two periods (i.e., before and after COVID-19), variables land use mix, born overseas, 5 years residency, CBD income, and count intersection exhibited positive signs for all local estimates. For both periods, the variables of average household size, distance to major roads, consistently exhibited negative signs for all the local estimates. Moreover, the signs of the following coefficients: distance to CBD, distance to PT, over 65 years of age, and population density were not consistent.

The proportion of people over the age of 65 had a positive correlation with crash frequency in the central and southern areas before COVID-19; this variable’s sign remained positive in the center, east, and northeast following COVID-19. Before COVID-19, population density was highly correlated with the probability of crashes for older adults in the central area. After COVID-19, the connection remained significant and seemed stronger in most regions of the metropolitan and central areas. Average household size had a negative relationship with crash frequency in all regions, and was greater in the northwest and south. Inhabitants with 5 years’ residency had a positive association with the risk of crashing before the pandemic, and the pattern after COVID-19 remained quite similar. Before and after the pandemic, born overseas had a negative link with crash frequency in the south, but a positive correlation in the remainder of the metropolitan area.

There was a weak association between crash frequency and proximity to public transit outside of the core, inner suburbs, but a negative one within the suburbs. For almost all metropolitan locations, the distance to major roads was negative, and there was minimal variation before and after COVID-19. The number of crashes increased as the degree of land use variety in a block increased.

This suggests that before the emergence of COVID-19, there were more crashes in the south-, east-, and west-central regions. Except for the southern region, this trend has been stable since the end of the COVID-19 period. Crash risk was positively correlated with the number of intersections per block, and this trend held true both before and after the pandemic. There was a negative, though weak, correlation between distance from the CBD and the frequency of crashes in the central region, inner suburbs, and the south, and a stronger correlation in the north. No observable change occurred following COVID-19. The positive link between CBD and income that existed before COVID-19 persisted after the virus was spreaded, with higher coefficients in the central and western areas.

Contradictory signs have been referred to as “the challenge with counterintuitive signs” (*59*). Multivariable multicollinearity may be a factor here. On a regional or even local scale, multicollinearity could have existed between some of the explanatory variables, however, this was not found to be the case. The intersection count variable had the highest explanatory power before COVID-19, and was highest in the central and northwestern regions (according to the top left map of Figure 4 (before COVID-19): Local R2 = 0.37). However, after COVID-19, its value reduced to 0.22 compared to before COVID-19.

**Figure 6.**
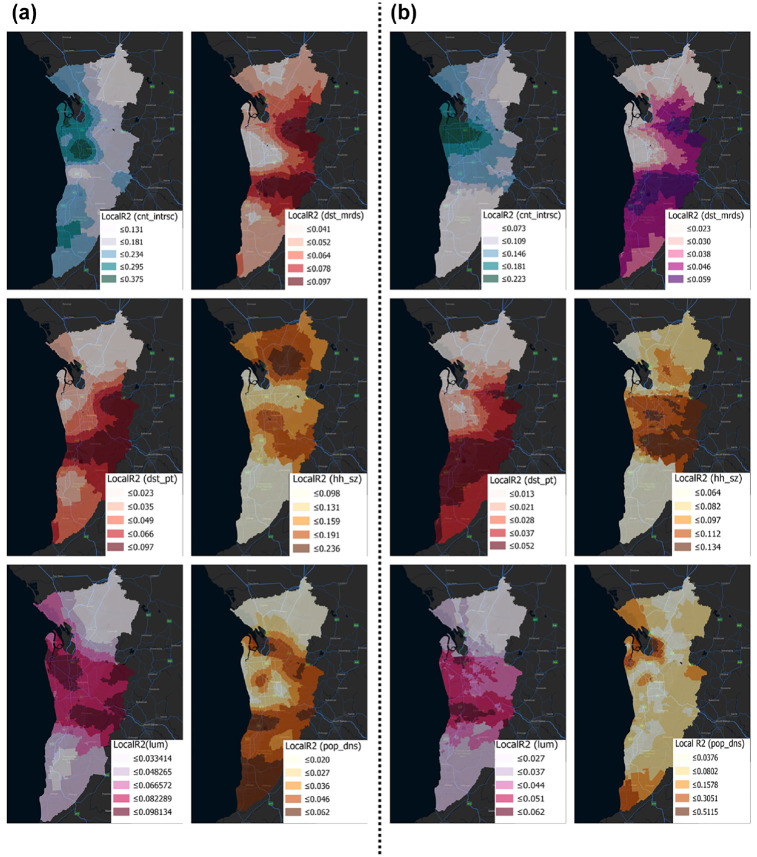
Local R2 indicators of significant factors affecting older adults’ crashes: (a) before and (b) after COVID-19.

The next significant variable based on the coefficients was household size, which was originally concentrated in the north. After COVID-19, the strength of this variable reduced from 0.23 to 0.13, and its hotspots shifted from the north to the center. This coefficient’s sign was negative. Population density was the third key variable to have a positive association. Following COVID-19, its explanatory power rose from 0.25 to 0.41. This was particularly prevalent in the southern, central, and northwestern areas. Blocks with greater population density were more likely to have more people and more activities, which could result in more collisions (*39*). Three variables including distance to PT, distance to major roads, and land use mix also had a significant impact on the crash frequency with a Local R2 ranging from 0.1 to 0.25. The rest of the factors had a modest geographic correlation with crash frequency (according to the top left map of Figure 5 (after COVID-19): Local R2 < 0.10) (Figure 6).

**Figure 7.**
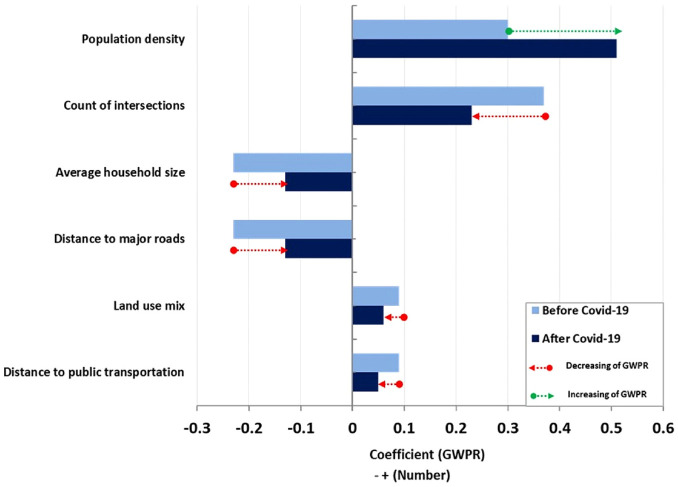
Signs and coefficients of the significant variables in the GWPR model, before and after COVID-19.

**Figure 8.**
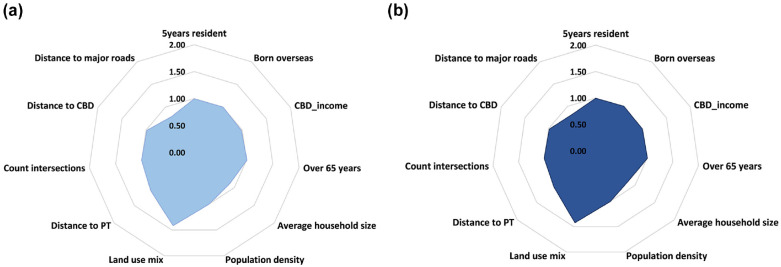
The IRR of variables in the NBR model: (*a*) before and (*b*) after COVID-19. Note: IRR = incident rate ratio; CBD = central business district; PT = public transport.

A summary of the signs and the significance of the variables’ impact on crash frequency is provided in Figure 7. This indicates the trends in Local R2 and the signs of the relationships between significant indicators and the significant changes in the number of older adults’ crashes before and after the pandemic. The figure demonstrates that the population density variable with an increasing Local R2 index and the count of intersections with decreasing Local R2 index had higher effects than other variables. Furthermore, the direction of the correlation index remained constant across all variables. Following these two variables, average household size and distance to major roads with a decrease in the Local R2 index, both before and after the pandemic, were the next priorities; their correlation signs remained unchanged after the pandemic. Figure 8 provides the IRR indicator, which indicated that the predicted change in crash frequency was mainly affected by land use mix.

## Discussion and Conclusions

As a result of COVID-19, the travel habits of Australians have shifted significantly, which has affected the number and severity of traffic crashes. Since the introduction of COVID-19 lockdowns, Australians have been taking more risks on the roadways as a result of decreasing travel volumes and congestion around the country (*60*). The present study evaluated accident data before and after COVID-19 to determine the impact of the pandemic on the frequency and location of crashes involving older people as a vulnerable segment of society. Several statistical and geographical approaches were used, and the impact of various sociodemographic and built-environment-related components were thoroughly investigated.

The preliminary results indicated a 19.5% reduction in the frequency of crashes after the pandemic compared with the 3-year average, which was surmised to be a direct effect of the COVID-19 lockdown travel restrictions rules. This investigation also found that the drop in crashes occurred on both a monthly and daily basis. The statistics indicated that during the first 3 months of the pandemic (i.e., April, May, and June 2020), there was a lower proportion of count crashes compared with the same 3-month period before the pandemic. The weekdays exhibited a similar pattern, with fewer crashes occurring on workdays (except on Wednesdays). Moreover, because of nightlife restrictions during the pandemic, the proportion of crashes occurring during daylight hours showed a significant increase. Moreover, although the reduction in traffic volume resulted in fewer crashes overall, the severity of crashes was slightly raised because some drivers felt able to travel at higher speeds (*61*).

The NBR results for both periods revealed the significant role of land use mix (*β* = 0.483), distance to major roads (*β* = –0.238), and distance to public transit (*β* = 0.082) as built environment and average household size (*β* = –0.114) as sociodemographic variables. The increase in the number of crashes involving people aged 65 and older in Adelaide was attributed to three main factors: an increase in pedestrian–vehicle conflicts in areas of mixed land use; proximity to crash high-risk corridors; and distance from public transit stations in areas controlled by traffic-calming strategies. Conversely, relying on younger family members to take care of some of the routine activities produced an opposite trend in crash frequency.

First, a key policy priority should therefore be to carefully analyze the temporal and spatial changing patterns of the crash count and severity data, as they appear to be strongly dependent on the time conditions and spatial characteristics of the environment. Owing to the greater vulnerability of older people, the results implied that in traffic management planning (*62*) their needs should be addressed first, proportional to the demographic demands of older individuals and their population density at the urban block level. Second, it is vital to consider variations in the built environment in relation to their impact on crashes in the central and in peripheral areas of metropolitan areas (*36*). For instance, the spatial distribution of activities and land uses, especially mixed land uses in the central part of metropolitan Adelaide (64–67), increased the trip frequency and, consequently, the probability of increasing the frequency of crashes. Third, improving the legibility of the urban environment by placing appropriate traffic signs, informing, warning, and controlling the speed by calming streets are among the essential policies for improving the visual knowledge, and understanding of people involved in crashes (*65*, *66*). Fourth, the pandemic’s positive experience in reducing the number of crashes owing to the reduction in physical conflicts and teleworking could represent management policies for new ways of employment through cyberspace in the future in Australia (*67*). To prevent unnecessary commuting, this would necessitate empowering the older (65+) population through “aging in place” policies (*68*), for instance increasing their digital literacy and facilitating online shopping, recreation, medical and health services, and employment options.

This study used a hybrid approach that incorporated geographically varying parameters into a conventional Poisson model, combining a statistical model based on a mathematical distribution with a local spatial regression model in each spatial unit to provide more powerful explanations for variations in crash patterns. This methodology can be applied in various settings and allows for improvements in the model structure. For instance, a semi-parametric GWPR can be used instead of the standard GWPR method. This modification enables restricted parameters to vary spatially despite the use of all variable parameters in the GWPR method (*69*).

The before-and-after comparisons in this study were susceptible to limitations that should be addressed in future research. Changes in traffic volumes and the ensuing shifts in crash rates (as opposed to mere shifts in crash frequency) must be taken into account whenever discussions of reductions or increases in crash frequency are undertaken. Traffic volume is often utilized as a covariate in regression models of crash frequencies, along with the length of the road segment under consideration. If the population’s exposure to risk has altered—especially considering here that traffic volumes dramatically decreased in the early months of the pandemic—then the purpose of comparing the periods before and after a crash may be questioned. Moreover, crash data supplied by police authorities often underestimate crashes, particularly for vulnerable road users.

Future research is advised to take into account the traffic volume of the road network and its influence on collision level, since this was a significant limitation of the current study. There was also lack of access to behavioral, personal, and psychological information about the older adults, which has been shown to be highly effective in explaining road crashes involving older adults (*70*). One of the demographic-related features of older drivers is their diminishing physical health and -resilience to serious injuries. As a result, age-related physical, visual, hearing, and cognitive limitations may limit one’s ability to carry out driving tasks and manage a range of complicated road scenarios, which are more prevalent in older driver crashes (*71*). Future research could address this gap by collecting personal information through direct surveys or focus groups. Because older adults have been shown to have varying levels of cognitive and mental abilities to face the risks associated with their travel behavior (*46*), a comparative analysis of crash occurrence among different segments of the older population, such as comparing younger groups with senior adults especially by controlling for reaction times (*72*, *73*), could be another interesting topic for future research. Moreover, the separation of work- and nonwork trips in the crash analysis is necessary (*74*) because the share of nonwork trips taken by older adults are greater than work trips, and this proportion is likely to have changed during the COVID-19 era (*75*). The analysis of crash patterns for various COVID-19 waves could also be significant. We also propose to explore how COVID-19 may have affected dangerous driving behaviors such as speeding, smartphone-, and alcohol use among drivers.
